# Manipulation of flowering time and branching by overexpression of the tomato transcription factor *SlZFP2*

**DOI:** 10.1111/pbi.12584

**Published:** 2016-06-29

**Authors:** Lin Weng, Xiaodong Bai, Fangfang Zhao, Rong Li, Han Xiao

**Affiliations:** ^1^ National Key Laboratory of Plant Molecular GeneticsCAS Center for Excellence in Molecular Plant SciencesInstitute of Plant Physiology and EcologyShanghai Institutes for Biological SciencesChinese Academy of SciencesShanghaiChina; ^2^ Center for RNA Molecular BiologyCase Western Reserve UniversitySchool of MedicineClevelandOHUSA

**Keywords:** RNA sequencing, overexpression, transcriptional regulation, flowering time, branching, tomato (*Solanum lycopersicum*)

## Abstract

Flowering of higher plants is orchestrated by complex regulatory networks through integration of various environmental signals such as photoperiod, temperature, light quality and developmental cues. In Arabidopsis, transcription of the flowering integrator gene *FLOWERING LOCUS T* (*FT*) that several flowering pathways converge to is directly regulated by more than ten transcription factors. However, very little is known about the transcriptional regulation of the *FT* homolog *SINGLE FLOWER TRUESS* (*SFT)* in the day‐neutral plant tomato (*Solanum lycopersicum*). Previously, we showed that the zinc finger transcription factor *SlZFP2* plays important roles in regulation of seed germination and fruit ripening in tomato and also found that overexpression of *SlZFP2* impacted flowering and branching. Here, we characterized in detail the early flowering and high branching phenotypes by overexpression of this transcription factor. Our data showed that overexpression of *SlZFP2* accelerated flowering in an *SFT*‐dependent manner as demonstrated by elevated *SFT* expression in the leaves and the transcription factor's binding ability to *SFT* promoter *in vitro* and *in vivo*. Furthermore, overexpression of the *SlZFP2* gene in the *sft* plants failed to rescue the mutant's late flowering. Through analysis of grafting phenotype, growth response of branches to auxin application and transcriptome profiling by RNA sequencing, we also showed that overexpression of *SlZFP2* affected shoot apical dominance through multiple regulatory pathways. Our results suggest that the transcription factor *SlZFP2* has potential applications in genetic modification of plant architecture and flowering time for tomato production and other crops as well.

## Introduction

The timing of flowering is crucial for higher plants to complete their life cycles in response to environmental conditions. Manipulation of flowering time has also great potential applications in plant breeding and is being under extensive investigation. In many plant species, flowering time is orchestrated by complex regulatory networks through integration of various environmental signals such as photoperiod, temperature, light quality and developmental cues. Several flowering pathways perceiving different environmental and developmental stimuli converge to few flowering integrators, for example, *FLOWERING LOCUS T* (*FT*), *SUPPRESSOR OF OVEREXPRESSION OF CONSTANS 1* (*SOC1*) and *AGAMOUS‐LIKE 24* (*AGL24*) in Arabidopsis (Michaels, 2009; Navarro *et al*., 2011; Pin *et al*., 2010; Tsuji *et al*., 2010). FT, mainly expressed in leaf, is transported to shoot meristems (SAM) where it interacts with FLOWERING LOCUS D (FD) to induce the transition of SAM to floral meristems (FM) (Wigge *et al*., 2005). At transcription level, *FT* is directly regulated by a number of transcription factors in response to different stimuli. For example, *FT* transcription is directly activated by CONSTANS (CO), CRYPTOCHROME‐INTERACTING BASIC–HELIX–LOOP–HELIX1 (CIB1), WRKY71, PHYTOCHROME‐INTERACTING FACTOR 4 (PIF4) and Morf‐related Gene 2 (MRG2) (Kumar *et al*., 2012; Liu *et al*., 2008, 2013; Tiwari *et al*., 2010; Xu *et al*., 2014; Yu *et al*., 2016). In addition, *FT* transcription is also directly repressed by TEMPRANILLO (TEM) 1 and 2, TARGET OF EAT (TOE) 1 and 2, SHORT VEGETATIVE PHASE (SVP), CYCLING DOF FACTOR1 (CDF1), EARLY‐FLOWERING MYB PROTEIN (EFM), SCHLAFMUTZE (SMZ) and SCHNARCHZAPFEN (SNZ) (Marin‐Gonzalez *et al*., 2015; Mathieu *et al*., 2009; Song *et al*., 2012; Yan *et al*., 2014; Zhang *et al*., 2015). Thus, transcriptional regulation of *FT* expression plays a crucial role in flowering time control in Arabidopsis.

Flowering is also regulated by phytohormones either through *FT* or other flowering regulators. For example, gibberellins (GAs) promote flowering through increasing *FT* expression in the vascular tissue under inductive long‐day condition (Porri *et al*., 2012). Abscisic acid (ABA), acting antagonistically with GA during seed germination, is also implied to play a role in regulation of flowering time (Wilmowicz *et al*., 2008). ABA‐deficient and insensitive mutants of Arabidopsis flower earlier under short‐day conditions (Martinez‐Zapater *et al*., 1994), indicating ABA has a repressive role in flowering time control. Some evidences show that flowering inhibition by ABA is likely through transcriptional regulation of the flowering repressor *FLOWERING LOCUS C* (*FLC*) because two ABA signalling components ABA INSENSITIVE 4 and 5 (ABI4 and ABI5) directly activate *FLC* transcription (Shu *et al*., 2016; Wang *et al*., 2013; ). However, under stress conditions, ABA may delay Arabidopsis flowering through GA pathway because in the quadruple‐*della* mutant, the transcription of *LEAFY* (*LFY*) was elevated (Achard *et al*., 2006), likely due to diminished DELLA repression on *LFY* and *SOC1* (Achard *et al*., 2007). On the other hand, *FT* seems to regulate stomatal opening and seed dormancy in Arabidopsis through ABA signalling pathway (Chen *et al*., 2014; Kinoshita *et al*., 2011). Nevertheless, there is an evident interconnection between ABA signalling and flowering pathways.

*SINGLE FLOWER TRUESS* (*SFT*), the homolog of *FT*, is a major player in flowering time control of the day‐neutral plant tomato (*Solanum lycopersicum*) (Lifschitz and Eshed, 2006; Lifschitz *et al*., 2006, 2014; Molinero‐Rosales *et al*., 2004). Functional conservation has also been revealed for the *FD* homolog *SUPPRESSOR OF SP*/*SP‐Interacting G‐BOX* (*SSP/SPGB*) and the flowering repressor *TERMINAL FLOWER1* (*TFL1*) homolog *SELF‐PRUNING* (*SP*) (Park *et al*., 2014; Pnueli *et al*., 1998). *SFT* and *SP* have additional functions in regulation of shoot architecture because mutations in the two genes cause either altered shoot growth pattern or reversion of inflorescence into leaves (Lifschitz *et al*., 2014). When *sp* mutation is present, *sft* heterozygosity exerts yield heterosis in an *SFT* dosage‐dependent manner (Jiang *et al*., 2013; Krieger *et al*., 2010). This indicates that transcription of *SFT* is tightly regulated. Despite its crucial roles in regulation of flowering and shoot architecture, how *SFT* is transcriptionally regulated in tomato is unclear. In addition to the above‐mentioned flowering genes, mutations in *UNIFLORA (UF)*,* Blind (Bl)*,* JOINTLESS (J)* and *COMPOUND INFLORESCENCE* (*S)* also affect flowering in tomato (Dielen *et al*., 2001; Quinet *et al*., 2006a,b, 2010). But the molecular mechanisms whereby these genes regulate flowering are unknown.

Plant architecture is largely determined by shoot branching. The formation of lateral shoots in tomato requires *Lateral suppressor* (*Ls*), *Bl* and its homologs *Bli1* and *Bli3* (Busch *et al*., 2011; Schmitz *et al*., 2002; Schumacher *et al*., 1999). After the formation of axillary meristems for developing lateral shoots, the outgrowth of shoot branches is regulated by auxin, cytokinins and strigolactones (Rameau *et al*., 2015). Auxin synthesized in shoot tips is associated with apical dominance; the apex‐derived auxin inhibits the growth of axillary buds below and its depletion from stem after decapitation releases bud dormancy. Because the movement of auxin in the main stem is directional—only moving downwards but not upwards into buds, the action of auxin on shoot branching is thought to be indirect (Muller and Leyser, 2011). Nevertheless, apical dominance is reduced in the auxin perception mutants of Arabidopsis *tir1* and *cul1* (Moon *et al*., 2007; Ruegger *et al*., 1998). As mentioned above, *Bl* is involved in flowering time control in tomato, its pepper homolog *CaBLIND* also regulates flowering in addition to shoot branching (Jeifetz *et al*., 2011). Moreover, genetic analysis of multi‐parent recombinant inbred lines (AMPRILs) in Arabidopsis has revealed that flowering time genes *FLC*,* FRIGIDA* (*FRI*) and *FT* have pleiotropic effects on shoot branching (Huang *et al*., 2013). Thus, these findings suggest that there are possible interconnections among the genetic networks regulating flowering and shoot branching.

Previously, we showed that overexpression of the zinc finger transcription factor *SlZFP2* affected multiple traits including flowering, branching, seed germination and fruit ripening (Weng *et al*., 2015). We demonstrated that *SlZFP2* regulates seed germination through direct transcription repression on ABA biosynthetic genes and it controls ripening by preventing *Colorless non‐ripening (CNR)* expression before the onset of ripening process, but how overexpression of *SlZFP2* affected flowering and branching was not addressed. In this study, by phenotypic, gene expression and biochemical analysis, we showed that the early‐flowering phenotype by overexpression of *SlZFP2* was resulted from elevated *SFT* transcription in the leaves, and the increased branching was due to weakened apical dominance. Moreover, gene expression analysis demonstrated that *SlZFP2* is required for *SFT* expression during fruit development. Because SlZFP2 directly binds to *SFT* promoter *in vivo* and *in vitro*, our study provides a promising strategy to manipulate flowering time for improvement of tomato production.

## Results

### Overexpression of *SlZFP2* promotes flowering in an *SFT*‐dependent manner

**Figure 1:**
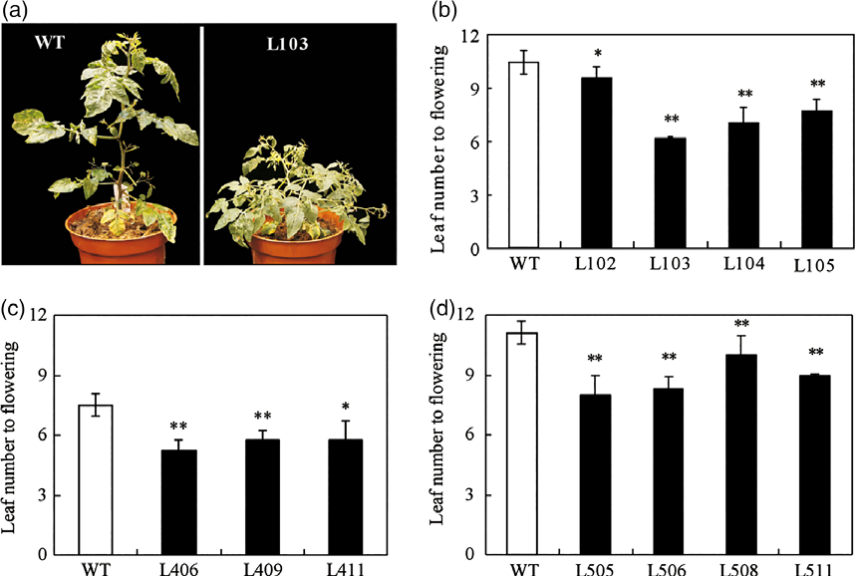
Overexpression of *SlZFP2* accelerates flowering. (a) A representative overexpression line of *HA‐SlZFP2* from *S. pimpinellifolium* LA1589 (L103) and the wild type (its nontransgenic sibling) showing the difference in flowering time. Photographs were taken at 45 days after germination when the wild‐type plants had only a small visible inflorescence, but the plants overexpressing *HA‐SlZFP2* had opened flowers. (b) Flowering time of four *HA‐SlZFP2* overexpression lines from LA1589 (L102–L105) and the wild type. Flowering time was quantified by leaf number before inflorescence formation. (c) Flowering time of three *HA‐SlZFP2* overexpression lines from M82 (L406, L409 and L411) and the wild type. (d) Flowering time of four *SlZFP2* overexpression lines from LA1589 (L505, L506, L508 and L511) and the wild type. Data (b–d) were mean ± SD from 8 to 10 plants. Statistical significance of *P*‐values was based on Student's *t*‐test. *P <* *0.05; **P <* *0.01.

We previously showed that the zinc finger protein *SlZFP2* negatively regulates ABA biosynthesis during fruit development and ripening (Weng *et al*., 2015). In addition to its role in regulation of seed germination and ripening, we also found that the transgenic plants overexpressing *SlZFP2* fused with HA tag (*HA‐SlZFP2*) or its coding sequence alone displayed (*SlZFP2*) early‐flowering phenotype (Figure 1a). To investigate the early‐flowering phenotype by overexpression of *SlZFP2* in more detail, we further quantified the flowering time of these overexpression lines in both genetic backgrounds of *Solanum pimpinellifolium* LA1589 and the cultivated tomato M82, respectively. The leaf number formed before the first inflorescence is predictable and consistent in a given growth condition, making it a good indicator for flowering time. In our growth conditions, the wild‐type (nontransgenic) plants of LA1589 and M82 form 11–12 and 7–8 leaves before the first inflorescence, respectively (Figure 1b–d). In contrast, three to four transgenic lines from LA1589 and M82 overexpressing either *HA‐SlZFP2* or *SlZFP2* had significantly fewer leaves formed before the first inflorescence; several transgenic lines from LA1589 and M82 produced only six or seven leaves before their first inflorescences formed (Figure 1). This indicates that overexpression of either *HA‐SlZFP2* or *SlZFP2* can effectively shorten flowering time in both of LA1589 and M82.

**Figure 2:**
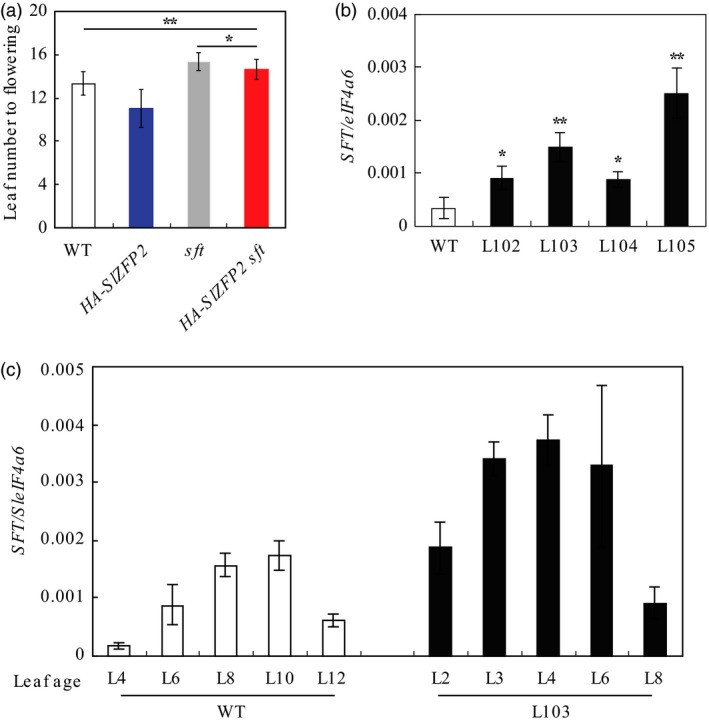
Overexpression of *HA‐SlZFP2* activates *SFT* expression in the leaves. (a) Overexpression of *HA‐SlZFP2* in the *sft* mutant (*HA‐SlZFP2 sft*) partially recapitulated its late‐flowering phenotype. n = 10. (b) *SFT* expression in the mature leaves of four *HA‐SlZFP2* overexpression lines from LA1589 and the wild type. (c) *SFT* expression in the leaves at different ages of the representative *HA‐SlZFP2* overexpression line L103. Total RNA was isolated from leaves of 45‐day‐old plants, *SFT* expression was determined by quantitative RT‐PCR, and data were presented as mean ± SD of three biological replicates. Developmental stages of leaves in (c) were indicated by the leaf numbers counting down from the youngest visible leaves. Statistical significance of *P*‐values was based on Student's *t*‐test. *P <* *0.05; **P <* *0.01.

Flowering time in tomato is mainly governed by *SFT* (Lifschitz *et al*., 2006; Shalit *et al*., 2009). It is plausible that overexpression of *SlZFP2* promotes flowering through *SFT* pathway. To test the possibility, we generated *sft* plants overexpressing *HA‐SlZFP2* by crossing between the *HA‐SlZFP2* overexpression lines and the *sft* mutant. Overexpression of *HA‐SlZFP2* in the *sft* mutant failed to rescue the mutant's late‐flowering phenotype to wild type, although it flowered earlier than the mutant did, but the effect was weak; the *sft* plants overexpressing *HA‐SlZFP2* formed 14.6 leaves on average before the first inflorescence, compared to 15.4 leaves of the *sft* mutant (Figure 2a). This suggests that promotion of flowering by overexpression of *SlZFP2* requires a functional *SFT* pathway. We then performed a quantitative reverse‐transcription PCR (qRT‐PCR) analysis of *SFT* expression in the mature leaves of four *HA‐SlZFP2* overexpression lines from LA1589 and the wild type. All the four overexpression lines had significantly higher *SFT* expression by more than twofolds (Figure 2b). Because *SFT* expression increases with leaf maturation (Lifschitz *et al*., 2006), we found overexpression of *HA‐SlZFP2* did not alter the temporal expression pattern of the florigen gene *SFT*; instead, it elevated its expression at all stages (Figure 2c). The results further support that overexpression of *SlZFP2* accelerates flowering through *SFT* pathway by activating its expression in the leaves.

**Figure 3:**
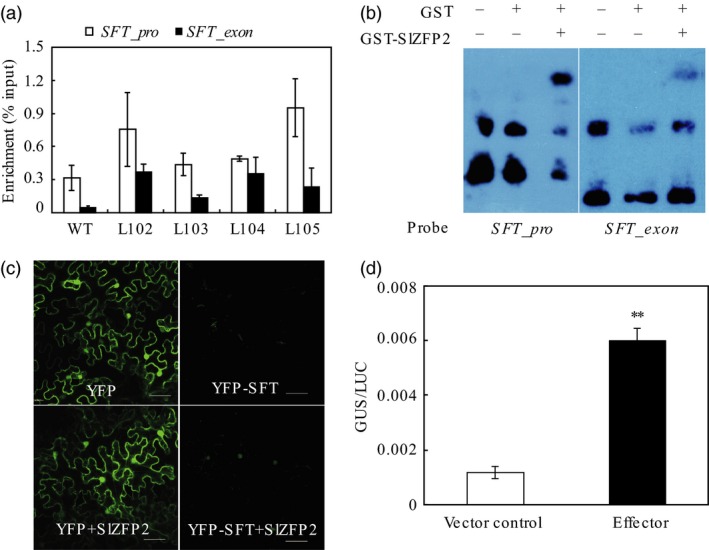
SlZFP2 binds to *SFT* promoter *in vitro*. (a) ChIP‐qPCR analysis of SlZFP2 binding to *SFT* chromosomal regions. ChIP was performed on co‐immunoprecipitated chromatins with HA antibody from leaves of the four *HA‐SlZFP2* overexpression lines and the wild type. (b) *In vitro* binding assay of GST‐SlZFP2 fusion protein to *SFT* chromosomal regions by EMSA. The two fragments used for EMSA were isolated from genomic DNA by the same set of primers used for ChIP‐qPCR in (a). (c) Activation of YFP‐SFT expression under the control of the native 2.0‐kb *SFT* promoter by SlZFP2 in the transiently transformed *N. benthamiana* leaves. Expression of YFP driven by the *35S* promoter was used as a control. (d) Activation of GUS expression under the control of the native 1.8‐kb *SFT* promoter by SlZFP2 in the transiently transformed *Arabidopsis* protoplasts. GUS activity was normalized by the luciferase (LUC) activity under the control of the *35S* promoter in the protoplasts co‐transformed with the effector (SlZFP2) or pUC118 (the vector control). Expression of *SlZFP2* (in c and d) was driven by the *35S* promoter. *SFT_pro*,*SFT* promoter; *SFT_exon*, exon region of *SFT*. Data were mean ± SD of three biological replicates. Statistical significance of *P*‐values was based on Student's t‐test. *P <* *0.05; **P <* *0.01.

Previously, we have shown that the transcription factor SlZFP2 recognizes cis‐elements containing (A/T)(G/C)TT motifs (Weng *et al*., 2015). *SFT* also contains multiple (A/T)(G/C)TT elements within its 1.5‐kb promoter region. Using HA antibody, we performed chromatin immunoprecipitation (ChIP) assay on the young leaves of four *HA‐SlZFP2* overexpression lines using the nontransgenic plants as wild‐type control. qPCR analysis of the ChIPed DNA revealed that the *SFT* promoter regions containing (A/T)(G/C)TT elements were enriched in the samples from the four *HA‐SlZFP2* overexpression lines and the bindings were further confirmed by EMSA using *Escherichia coli* expressed GST‐SlZFP2 on the same regions (Figure 3a,b). We further conducted a transient gene expression analysis to test whether high *SlZFP2* expression can enhance *SFT* transcription in tobacco leaves. Indeed, the expression of YFP‐SFT under the control of the 2.0 kb native *SFT* promoter was activated in *Nicotiana. benthamiana* leaves transiently overexpressing *SlZFP2* under the control of the 35S promoter (Figure 3c). Moreover, GUS expression driven by the 1.8 kb *SFT* promoter was increased dramatically in *Arabidopsis* protoplasts by overexpression of *SlZFP2* (Figure 3d). Thus, SlZFP2 has transcriptional activation activity to enhance *SFT* expression *in vitro* and *in vivo* and it can directly bind to the (A/T)(G/C)TT elements of the *SFT* promoter.

### Overexpression of *SlZFP2* increased branching

**Figure 4:**
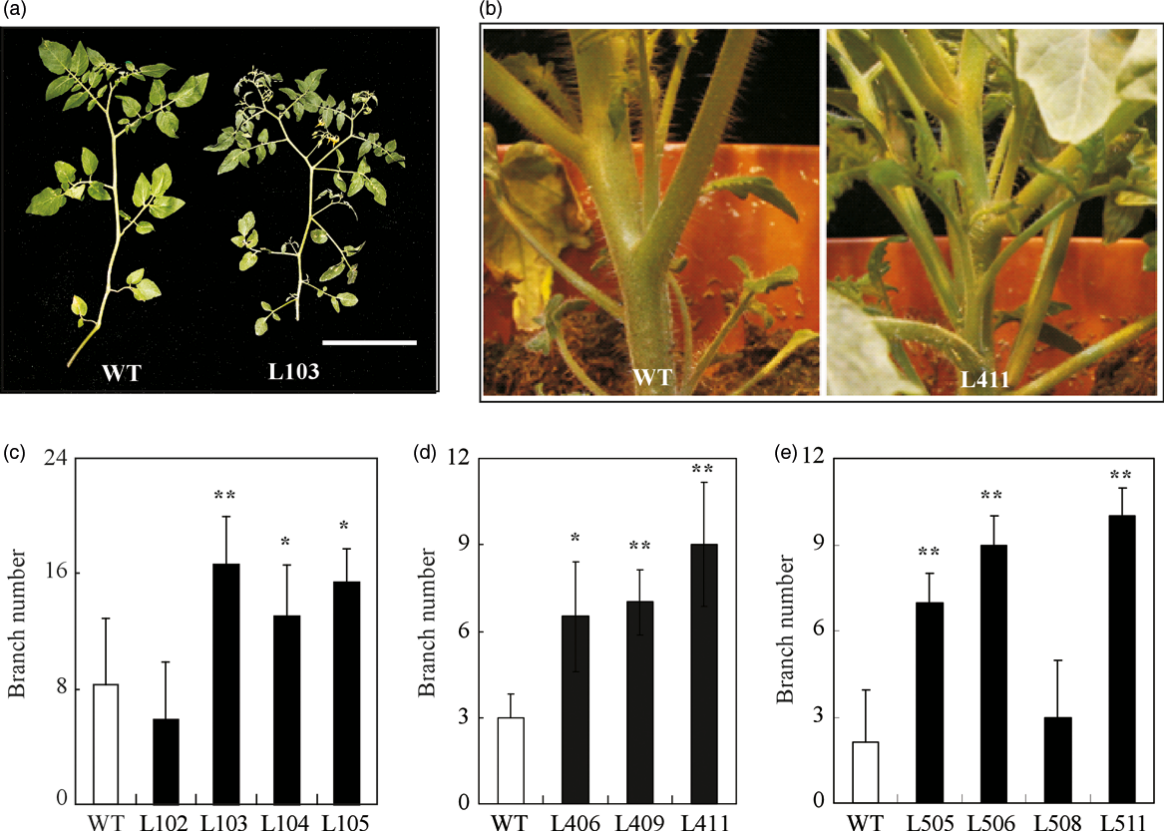
Overexpression of *SlZFP2* increases branching. (a) Branch images of a representative *HA‐SlZFP2* overexpression line from LA1589 (L103) and the wild type. (b) Images of a representative *HA‐SlZFP2* overexpression line from M82 (L411) and the wild type. (c) Branch numbers of four *HA‐SlZFP2* overexpression lines in LA1589 background and the wild type. (d) Branch numbers of three *HA‐SlZFP2* overexpression lines from M82 and the wild type. (e) Branch numbers of four *SlZFP2* overexpression lines in LA1589 background and the wild type. Data were mean ± SD, n = 4–15. Statistical significance of *P*‐values was based on Student's *t*‐test. *P <* *0.05; **P <* *0.01. Scale = 1 cm.

In addition to early flowering, we also observed high branching or fast bud outgrowth phenotype in these transgenic lines overexpressing *HA‐SlZFP2* or *SlZFP2* during vegetative development (Figures 1a and 4a,b; Weng *et al*., 2015). We quantified the numbers of branches formed on one‐and‐half‐month‐old plants overexpressing *HA‐SlZFP2* or *SlZFP2* in LA1589 and M82 backgrounds. Except for the line L102 from LA1589, branch numbers were significantly increased in the other three *HA‐SlZFP2* overexpression lines (Figure 4c,d). In addition, three of the four *SlZFP2* overexpression lines also had more branches (Figure 4e). Furthermore, the transgenic plants overexpressing *HA‐SlZFP2* produced side‐shoots much earlier than the wild type did on both of the main shoots and branches (Figure 4a). The results suggest that overexpression of *HA‐SlZFP2* or *SlZFP2* impacts bud outgrowth in both of LA1589 and M82.

**Figure 5:**
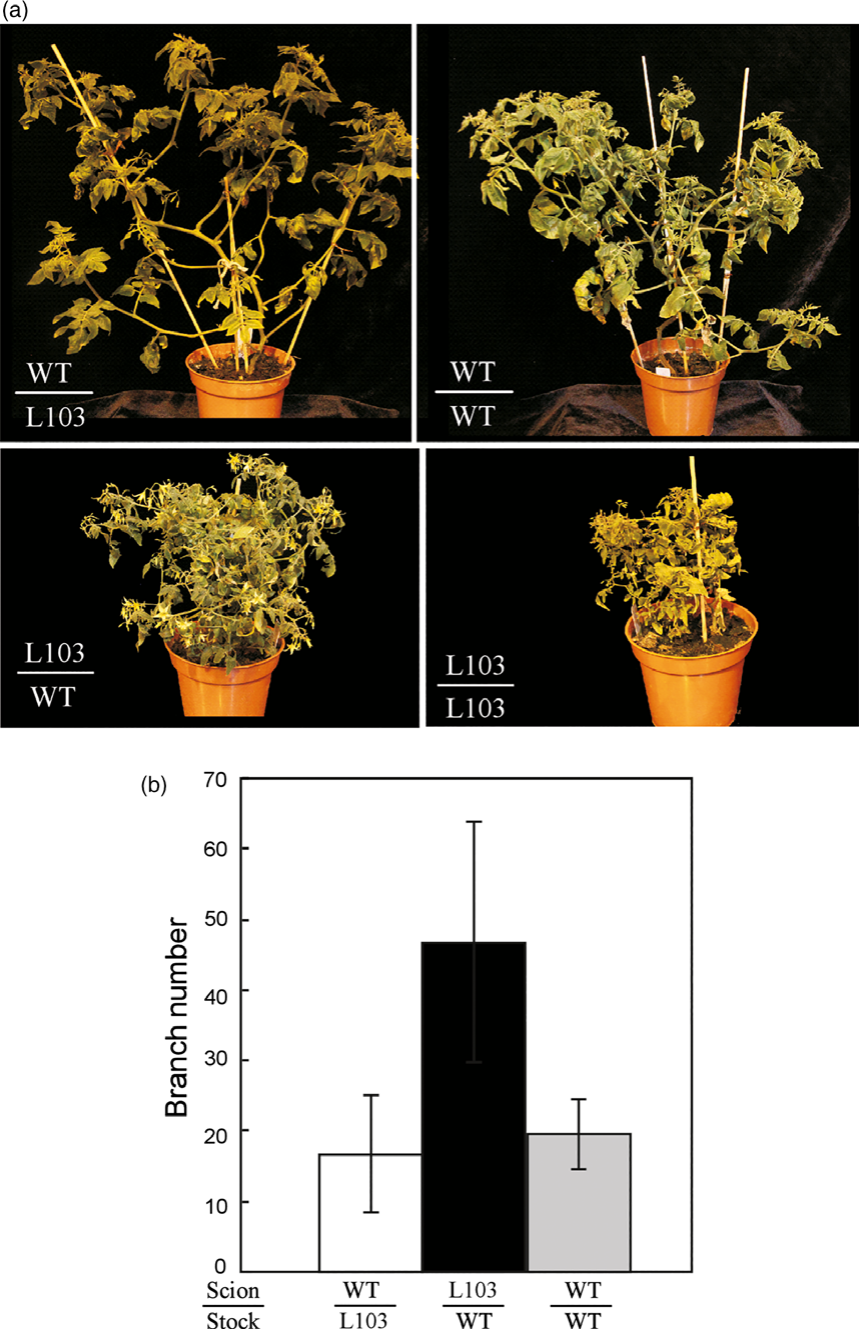
Grafting between the seedlings of the *HA‐SlZFP2* overexpression line and the wild type. (a) Images of grafted plants. (b) Branch numbers of grafted shoots. A representative *HA‐SlZFP2* overexpression line L103 was used for grafting. Due to slow and weak recovery after grafting, branch number was not recorded for transgenic shoots grafted on the rootstocks of the same genotype. Data were mean ± SD, n = 3–4.

The outgrowth of shoot branches is regulated by plant hormones including auxin, cytokinin and strigolactones (Rameau *et al*., 2015). Strigolactones are mainly synthesized in roots and transported to axillary buds to inhibit their outgrowth. To determine whether the increased branching phenotype observed on these transgenic plants overexpressing either *HA‐SlZFP2* or *SlZFP2* was caused by impaired strigolactone biosynthesis in the roots, we performed reciprocal grafting between the seedlings of the *HA‐SlZFP2* overexpression line L103 and the wild type. The wild‐type rootstocks did not suppress the branching of the scions overexpressing *HA‐SlZFP2*; they still produced more branches than the wild‐type scions grafted on the rootstocks of either L103 or the wild type (Figure**** 5). Self‐grafted shoots of the *HA‐SlZFP2* overexpression line recovered slowly, but the plant stature looked very similar with its nongrafted plants. The slow recovery of self‐grafted plants overexpressing *HA‐SlZFP2* was likely due to its less developed root system as shown previously (Weng *et al*., 2015). Nevertheless, the grafting results suggest that the high branching phenotype by overexpression of *HA‐SlZFP2* is likely independent of strigolactone biosynthetic pathway.

**Figure 6:**
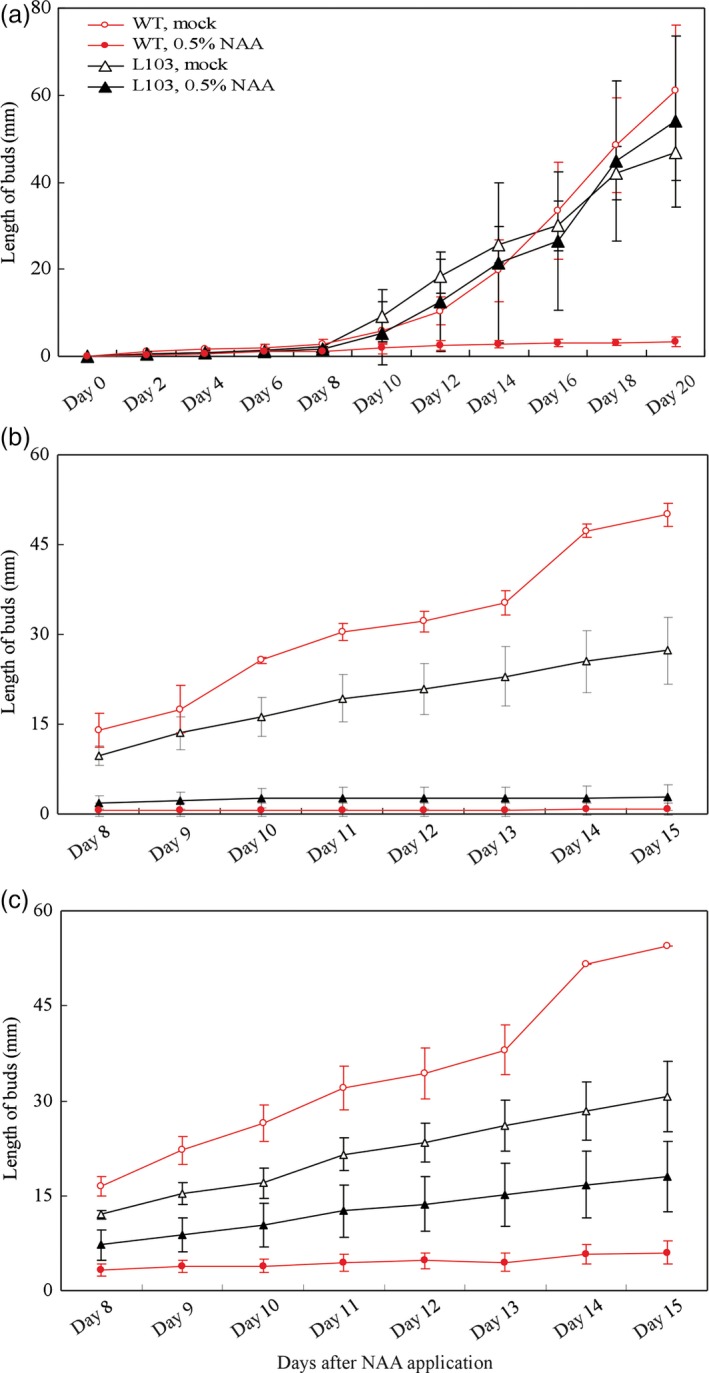
Growth response of the shoot branches overexpressing *HA‐SlZFP2* to auxin applied on the decapitated stumps. (a) Growth response of the second branch of the *HA‐SlZFP2* overexpression line L103 and the wild type to NAA application on decapitated stumps of young plants. (b) Growth response of the first branch of the *HA‐SlZFP2* overexpression line L103 and the wild type to NAA application on decapitated stumps of adult plants. (c) Growth response of the first branch of the *HA‐SlZFP2* overexpression line L103 and the wild type to NAA application on decapitated stumps of adult plants. The assay in (a) was conducted on six to nine plants at 15 days postgermination and those in (b and c) were conducted on five plants at 30 days postgermination. 0.5% (w/w) NAA in lanolin or lanolin only (mock) was applied to the decapitated stumps. Data were presented as mean ± SD.

In addition to root‐derived strigolactones, the growing shoot apex may inhibit the activation and outgrowth of axillary buds formed below, and the apical dominance is classically linked to shoot apex‐derived auxin (Leyser, 2005; Muller and Leyser, 2011; Teichmann and Muhr, 2015). We then tested the possibility that overexpression of *SlZFP2* weakens apical dominance by monitoring the growth response of the first and second branches below the decapitation site to NAA application. We first measured the branch growth response using 15‐day‐old plants of the *HA‐SlZFP2* overexpression line L103 and the wild type. As the first branches were completely suppressed, only the length of second branches was measured. Application of 0.5% NAA to the decapitated stumps almost completely suppressed the outgrowth of the second branches of the wild‐type plants. In contrast, the branch outgrowth of the *HA‐SlZFP2* overexpression line was not inhibited by 0.5% NAA (Figure 6a). Next, we tested the auxin sensitivity of branch outgrowth using relatively older plants of 30 days old. Without NAA applied on the decapitated stumps, the first and second branches of the *HA‐SlZFP2* overexpression line L103 from 30‐day‐old plants grew much slower than the wild‐type branches did (Figure 6b,c). When 0.5% NAA was applied, the growth of the first branch was similarly effectively inhibited in both L103 and the wild type (Figure 6b). However, NAA application had weaker inhibitory effect on the outgrowth of the second branch of the *HA‐SlZFP2* overexpression line (Figure 6c). These results imply that the branch outgrowth of the *HA‐SlZFP2* overexpression lines is less sensitive to auxin. Therefore, overexpression of *HA‐SlZFP2* likely weakens apical dominance.

### Transcriptional regulation by *SlZFP2*

To further understand how overexpression of *SlZFP2* increases branching and accelerates flowering at the transcription level, we conducted an RNA‐seq analysis on the shoot apices of two *HA‐SlZFP2* overexpression lines (L103 and L104) and the wild type (nontransgenic siblings from the two respective overexpression lines). RNA‐seq libraries were made of total RNA isolated from the shoot apices with small visible leaves from 45‐day‐old plants. After sequencing, the reads were mapped to the tomato reference genome (version SL2.4) by Tophat (Trapnell *et al*., 2009). After the uniquely mapped reads were assembled by Cufflinks (Trapnell *et al*., 2010), differentially expression genes (DEs) between the two *HA‐SlZFP2* overexpression lines and the wild type were first selected using a cut‐off of adjusted *P*‐value at 0.05 and fold change of 1.5. In addition, genes with expression changes larger than twofolds in the two lines and FPKM ≥0.1 were also considered to be differentially expressed. In total, we identified 707 and 569 genes, respectively, up‐regulated and down‐regulated by overexpression of *HA‐SlZFP2* (Table S1).

**Figure 7:**
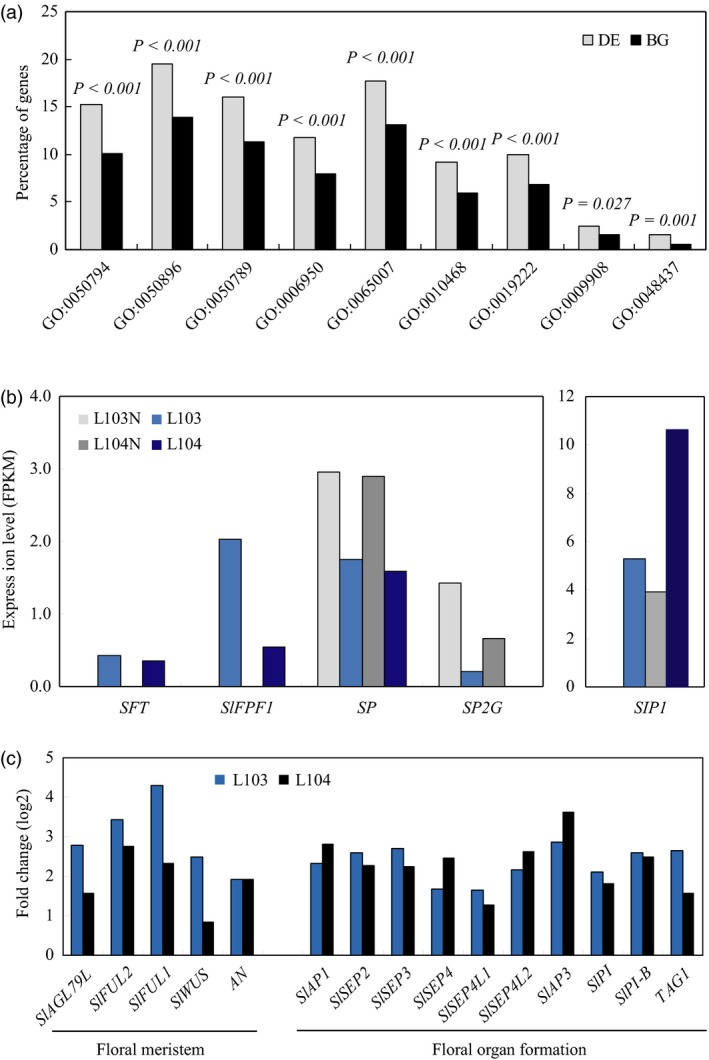
Differentially expressed genes involved in flowering time control and floral development. (a) Gene ontology analysis of differentially expressed genes between the two *HA‐SlZFP2* overexpression lines (L103 and L104) and the wild type. The annotated tomato genome contains 34,727 genes (http://solgenomics.net/, ITAG2.3 version), 23 237 genes had FPKM ≥0.1 in any of the four samples assayed and were arbitrary considered as expressed and were used as background in gene ontology analysis. Enrichment analysis of gene ontology terms was performed with agriGO toolkits on the 1276 genes regulated by *SlZFP2* in the shoot apex. Single enrichment analysis (SEA) was conducted using statistical method of hypergeometric in combination with Bonferroni multitest adjustment. Enriched GO terms against background with adjusted *P*‐value less than 0.05 were selected. (b) Transcript levels of flowering regulators. *SlFPF1*,* Solyc01 g066970*;*SP2G, Solyc02 g079290*;*SIP1*,* Solyc11 g007880*. (c) Fold changes of transcript levels for genes involved in floral development. Fold changes of expression levels were expressed in log2 ratios. GO:0050794, regulation of cellular process; GO:0050896; response to stimulus; GO:0050789, regulation of biological process; GO:0006950, response to stress; GO:0065007, biological regulation; GO:0010468, regulation of gene expression; GO:0019222, regulation of metabolic process; GO:0009908, flower development; GO:0048437, floral organ development.

Consistent to the role of *SlZFP2* in regulation of ABA pathway (Weng *et al*., 2015), the GO ontology analysis of DEs revealed that genes involved in stress responses were significantly enriched (Figure 7a). In addition, function category of floral development was also over‐represented. Particularly, *SFT* transcripts were only detected in the two overexpression lines, further confirmed that overexpression of *HA‐SlZFP2* induces *SFT* expression. *SlFPF1*, the putative homolog of Arabidopsis flowering promoter *FPF1*, was also only detected in the two overexpression lines, whereas the flowering repressors *SP* and *SELF‐PRUNING 2G* (*SP2G*), the putative ortholog of Arabidopsis *MOTHER OF FT* (*MFT*), were down‐regulated (Figure 7b). In addition, transcription of *self‐pruning interacting protein 1* (*SIP1*) was drastically elevated. Likely due to the elevated *SFT* and *SlFPF1* expression, genes involved in floral meristem formation were activated in the two overexpression lines, which then led to transcriptional activation of genes involved in floral organ formation (Figure 7c).

Auxin is mainly synthesized in shoot apex and its downward transport is believed to repress the outgrowth of axillary buds. The hormone is perceived by the SCF^TIR1^‐SKP1‐CUL protein complex (Salehin *et al*., 2015). Mutation in the auxin receptor gene *TIR1* of Arabidopsis leads to weakened apical dominance, and the *cul1* mutant is also short and bushy (Moon *et al*., 2007; Ruegger *et al*., 1998). Overexpression of *HA‐SlZFP2* down‐regulated *Solyc01 g067200* and *Solyc05 g009260,* encoding proteins showing high similarity with CUL1 and TIR1, respectively (Table S1). This suggests that overexpression of *HA‐SlZFP2* likely attenuated auxin signalling in the shoot apex. Furthermore, *Solyc08 g016060*, the *SPIKE1* (*SPK1*) homolog, which its mutation has been shown to induce PIN2 internalization in Arabidopsis (Lin *et al*., 2012), was also repressed in the two overexpression lines. However, the weakened apical dominance of the *HA‐SlZFP2* overexpression lines may only be explained partially by the down‐regulated expression of these auxin‐related genes because another half of 16 DE genes involved in auxin pathway were up‐regulated by overexpression of *HA‐SlZFP2*. The up‐regulated genes by overexpression of *HA‐SlZFP2* include auxin biosynthetic gene *Solyc09 g091090* (*YUC3* homolog), *Solyc01 g111640* (*SKP1* homolog) and six auxin response genes. In addition, the accelerated branch outgrowth in these *HA‐SlZFP2* overexpression lines may also be resulted from down‐regulated expression of the *Blind like 3* (*Bli3*) gene, encoding a MYB transcription factor that has been shown to regulate shoot branching (Busch *et al*., 2011).

## Discussion

Shoot branching involving initiation of axillary meristems, bud development and outgrowth is regulated by complex interaction of plant hormones and transcription factors. In tomato, the MYB transcription factor *Bl* and the GRAS family member *Ls* regulate axillary meristem formation (Schmitz *et al*., 2002; Schumacher *et al*., 1999). Overexpression of *HA‐SlZFP2* did not affect *Bl* and *Ls* expression, but down‐regulated the expression level of the *Bl* homolog *Bli3*. The down‐regulated *Bli3* may attribute, at least in part, to the branching phenotype in these *SlZFP2* overexpression lines because increasing branching phenotype was observed in its RNAi lines (Busch *et al*., 2011). However, genes involved in auxin, BR and cytokinin pathways were also impacted in the shoot apices of the *HA‐SlZFP2* overexpression lines, suggesting that the increased branching phenotype caused by overexpression of *SlZFP2* may also be related to impaired hormone signalling and/or crosstalks among these hormones. As the branch outgrowth of the *HA‐SlZFP2* overexpression lines was less sensitive to NAA application to the decapitated stumps, overexpression of *HA‐SlZFP2* likely weakened apical dominance. In agreement with the notion, the expression of the two genes encoding homologs of Arabidopsis TIR1 (*Solyc05 g009260*) and CUL1 (*Solyc01 g067200*) were down‐regulated in the shoot apices of the *HA‐SlZFP2* overexpression lines. It has been shown that loss‐of‐function mutations in the two genes caused weak apical dominance in Arabidopsis. Therefore, it is plausible that the weakened apical dominance was resulted from transcriptional repression of auxin signalling involved in regulation of bud outgrowth. Nevertheless, overexpression of *SlZFP2* increases branching likely independent of the strigolactone biosynthetic pathway as demonstrated by reciprocal grafting between the *HA‐SlZFP2* overexpression lines and the wild type.

*SFT* and *SP* are two key flowering regulators in tomato (Lifschitz *et al*., 2014). Our results demonstrated that overexpression of *SlZFP2* accelerates flowering mainly through activation of *SFT* expression, not by transcriptional repression of *SP*. Although *SP* expression was significantly down‐regulated in the shoot apices of the two *HA‐SlZFP2* overexpression lines, there was no obvious defect in sympodial shoot formation observed on any of these *SlZFP2* overexpression lines. Instead, the flowering phenotype of the plants overexpressing *HA‐SlZFP2* or *SlZFP2* resembles the *SFT* overexpression lines; the extreme early‐flowering lines overexpressing *HA‐SlZFP2* produced similar leaf number before the first inflorescence as the *SFT* overexpression lines did. EMSA and ChIP assay as well as transient gene expression analysis further confirmed that SlZFP2 activated *SFT* expression through direct binding to its promoter region containing (A/T)(G/C)TT element *in vivo* and *in vitro*. Thus, *SFT* is likely a direct target of SlZFP2. However, *SlZFP2* unlikely plays a major role in flowering time control mediated by *SFT* because suppressing this transcription factor by RNAi did not delay flowering (Weng *et al*., 2015). The notion is further supported by the different expression patterns between the two genes; *SlZFP2* is expressed in young leaves and shoot apex during vegetative development, whereas *SFT* is mainly expressed in mature leaves. Although *SFT* is unlikely a direct target of SlZFP2 in the leaves, this transcription factor regulates *SFT* expression in the fruit. By qRT‐PCR analysis, we found that in the wild‐type *SFT* was not only expressed in leaves but also during fruit development and its expression reached its maximal level at breaker stage when the fruit started to ripen and seed maturation was almost completed (Figure S1a). Overexpression of *HA‐SlZFP2*, like in leaves, activated *SFT* expression in mature green fruits, whereas down‐regulated *SlZFP2* expression by RNAi dramatically decreased *SFT* expression (Figure S1b,c). Although the role of *SFT* in fruit development remains to be unravelled, we speculate that during fruit development, the activation of the florigen gene by *SlZFP2* might be involved in fruit and/or seed development because we previously demonstrated that *SlZFP2* is required for fruit ripening and seed development (Weng *et al*., 2015). In Arabidopsis, it has been shown that FT can store the temperature memories in the fruit to control progeny's seed dormancy (Chen *et al*., 2014). It is plausible that *SFT* has similar function in tomato seed development because high expression of *SFT* was detected at breaker stage during fruit development. Furthermore, there are increasing evidences indicating that *FT* or its homologs in other species regulates diverse developmental processes other than flowering including lateral shoot outgrowth and stomatal opening in Arabidopsis as well as the formation of potato tuber and onion bulb (Lee *et al*., 2013; Navarro *et al*., 2011).

The transcription factor SlZFP2 is a negative regulator of ABA biosynthesis; overexpression of *SlZFP2* decreases ABA production (Weng *et al*., 2015). When compared with the *sft* mutant, the *sft* plants overexpressing *HA‐SlZFP2* flowered slightly but substantially earlier, indicating that high *HA‐SlZFP2* expression likely affected other flowering pathway independent of *SFT*. One explanation for this observation is that the suppressed ABA biosynthesis by overexpression of *HA‐SlZFP2* promoted early flowering because the tomato ABA‐deficient mutant *sitiens* (*sit*) and *flacca* (*flc*) also flowered slightly earlier; the two mutants formed fewer leaves before the first inflorescence occurred (Figure S2a). The result is consistent with previous observations that Arabidopsis ABA‐deficient and insensitive mutants flower earlier (Martinez‐Zapater *et al*., 1994). Recently, it has been shown that ABA delays flowering in Arabidopsis through transcriptional regulation of the *FLC* gene mediated by *ABI4* and *ABI5* (Shu *et al*., 2016; Wang *et al*., 2013). The repressed *FLC* expression releases its inhibition on *FT* transcription to induce flowering. It is unclear that tomato has a functional *FLC* pathway although there are members of MADS genes sharing high similarity with FLC. But, the action of ABA on tomato flowering time control is complex because no early‐flowering phenotype was observed on the ABA‐deficient mutant *notabilis* (*not*) and the flowering time of these mutants was not well associated with *SFT* expression in the leaves (Figure S2b).

Developing new elite crop varieties with optimal flowering time and plant architectures is a prerequisite to meet the increasing demand for food, feed and biofuel production. For many crops, the transition of vegetative to reproductive phase is governed by the conserved FT pathway, and natural allelic variations at *FT* orthologs have been widely used in traditional breeding programmes (Blumel *et al*., 2015). Thus, manipulating the transcription of these *FT* orthologs has great potential applications in breeding programmes of crops whose flowering time is mainly governed by this conserved pathway. The transcription factor SlZFP2 has the activity to activate the expression of the tomato *FT* homologous gene *SFT* by direct binding to the core (A/T)(G/C)TT elements in the latter's promoter region, making it an excellent candidate to manipulate *SFT* expression for flowering time control in tomato. In addition, it has also been shown that *SFT* not only controls flowering time but also regulates heterosis depending on the presence of a mutation in *SP* (Krieger *et al*., 2010). The yield heterosis observed in heterozygous *sft* hybrids is likely due to dosage‐dependent *SFT* action on shoot and inflorescence development resulting from the modification of flowering time because reducing *SFT* expression by artificial microRNA recapitulated the shoot architecture phenotypes of *sft/+* heterozygotes (Jiang *et al*., 2013). It has been proposed that meristem‐specific SFT/SP ratio mediates plant growth balance, which high ratio causes growth arrest and termination in shoot meristems (Lifschitz *et al*., 2014). In the genetic background without the *sp* mutation such as LA1589, overexpression of either *HA‐SlZFP2* or *SlZFP2* has no obvious negative impact on fruit weight (Weng *et al*., 2015). Thus, manipulation of *SlZFP2* expression might also be used for yield improvement through optimizing the balance between growth and flowering in tomato and other crops.

## Experimental procedures

### Plant materials and growth conditions

The wild relative of tomato *Solanum pimpinellifolium* LA1589, the mutants *flc*,* sit*,* not*,* sft* and the cultivars Ailsa Craig, LA0534 and LA0535 used in this study were obtained from Tomato Genetics Resource Center at University of California, USA. The tomato cultivar M82 was provided by Dr. Daniel Zamir at the Hebrew University of Jerusalem, Israel. Generation of the transgenic lines overexpressing *HA‐SlZFP2* or *SlZFP2* in LA1589 and M82 as well as the *SlZFP2* RNAi lines from LA1589 has been described in our previous study (Weng *et al*., 2015). Because most of the M82 lines overexpressing *HA‐SlZFP2* had no or very few seeds, we conducted most of the experiments described in this study on the *HA‐SlZFP2* overexpression lines from LA1589. The transgenic lines together with the wild‐type plants were grown in phytotron under 70–80% relative humidity at 20–25 °C and illuminated for 16 h daily by light intensity of 150 mE/m^2^/s from metal halide lamps and high pressure sodium lamps. To maintain optimal growth, the three ABA‐deficient mutants (*not, sit* and *flc*) were sprayed 50 μm ABA at 10‐day intervals. Plants were fertilized weekly with all‐purpose fertilizer and watered as needed.

### Phenotypic analysis of *SlZFP2* overexpression lines

Flowering time and branch number was recorded mainly at 45 days postgermination on 3–4 independent transgenic lines and their corresponding siblings segregated from heterozygous transgenic lines (using as the wild‐type controls). Flowering time was recorded as leaf number before first inflorescence. For branching measurements, only branches of 0.5 cm or longer were counted. Flowering time and branching phenotype were recorded for three to four independent transgenic lines overexpressing *HA‐SlZFP2* in LA1589 and M82 as well as four lines overexpressing *SlZFP2* in LA1589.

For apical dominance analysis, 15‐ and 30‐day‐old plants of the representative *HA‐SlZFP2* overexpression line L103 and the wild type were decapitated, and 0.5% (w/w) NAA mixed with lanolin was applied to the decapitated stumps. Same amount of lanolin without NAA was used as mock control. The lengths of the first and second branches below the decapitated sites were measured daily after treatment.

To test whether the high branching phenotype observed on these *SlZFP2* overexpression lines was caused by impacted strigolactone biosynthesis, reciprocal grafting was made between 15‐day‐old seedlings of the representative *HA‐SlZFP2* overexpression line L103 and the wild type and the number of branch (≥0.5 cm) was counted at 50 days after grafting.

### Profiling of global gene expression by RNA sequencing

Two homozygous transgenic lines L103 and L104 showing very similar phenotypes in flowering and branching were chosen for profiling gene expression regulated by *SlZFP2* via RNA sequencing (RNA‐Seq). Their respective nontransgenic siblings (L103N and L104N) were served as controls. Total RNA was extracted by Trizol reagent (Invitrogen, Carlsbad, California, USA) from shoot tips including one small visible leaf was collected from one‐and‐half‐month‐old plants as described previously (Xiao *et al*., 2009). Paired‐end sequencing libraries were created and sequenced by Shanghai Hanyu Bio on Illumina's Genome Analyzer IIx system using 100‐bp reads. Reads were mapped to tomato genome using Tophat program v.1.3.2 (Trapnell *et al*., 2009), and the transcripts were assembled using cufflinks (Trapnell *et al*., 2010) based on transcripts predicted in SL2.40 assembly from SGN (version ITAG2.3). In total, the numbers of fragments (one or both of the paired‐end reads) mapped to SL2.40 assembly were 13 560 517 (90.4%) for L103, 13 778 522 (91.9%) for L104, 13 592 147 (90.6%) for L103N and 13 834 382 (92.2%) for L104N.

The expression values of the transcripts were calculated in fragments per kilobase of transcript per million mapped reads (FPKM) (Mortazavi *et al*., 2008). Differentially expressed genes were identified using *t* statistics with *P* values adjusted for false discovery rate using Benjamini and Hochberg method (Benjamini and Hochberg, 1995). Adjusted *P* value of 0.05 or less was considered as statistically significant. Total of 1205 differentially expressed genes were identified. We verified transcript levels of 33 genes with different FPKM values by real‐time quantitative RT‐PCR (qRT‐PCR) on the same batch of RNA samples used for RNA‐seq and found the two methods are comparable (Table S2). For qRT‐PCR, transcripts level of genes with FPKM less than 0.2 was barely detected (CT >35). Therefore, the differentially expressed gene list was further filtered with arbitrary FPKM cut‐off at 0.1 and fold change at 1.5. Genes with twofold or higher change but not identified by *t* statistics were also included. In total, 1276 genes were considered differentially expressed between the *HA‐SlZFP2* overexpression lines and nontransgenic controls.

The raw reads and gene expression data have been deposited in the National Center for Biotechnology Information under accession number GSE45243.

### Real‐time quantitative RT‐PCR

Total RNA was extracted from various tomato tissues as described above. Residual genomic DNA in RNA samples was removed by RNase‐free DNase (New England Biolabs (Beijing), Beijing, China) at 37 °C for 10 min, and 1 μg of DNase‐treated total RNA was converted to first‐strand cDNA using First Strand cDNA Synthesis Kit (Fermentas, Vilnius, Lithuania, EU). qRT‐PCR was performed in three biological replicates using SYBR® Premix ExTaq™ (Takara DaLian, China) on an ABI Applied Biosystems StepOnePlus machine, except the data shown in Table S2 were from three technical replicates. Transcript level was expressed as relative expression normalized with *SleIF4*α*6* signals.

### Chromatin immunoprecipitation assay

Chromatin immunoprecipitation assay was performed on the four *HA‐SlZFP2* overexpression lines as described previously (Weng *et al*., 2015). Essentially, young leaves with shoot apices of 45‐day‐old plants were used for chromatin isolation. Chromatin was immunoprecipitated with HA monoclonal antibody (Sigma St. Louis, Missouri, USA) coupled on Dynabeads Protein G (Invitrogen, Carlsbad, California, USA). Eluted DNA samples were further purified by phenol/chloroform extraction after Protease K treatment. Then, 2 μL ChIPed DNA per sample dissolved in 100 μL TE was used for qPCR analysis.

### Electrophoretic mobility shift assay

Electrophoretic mobility shift assay (EMSA) was performed on *E. coli* expressed GST‐SlZFP2 fusion protein and DNA fragments of *SFT* promoter containing putative binding sites for SlZFP2 as described previously (Weng *et al*., 2015). The same primers used for ChIP‐qPCR analysis were used for PCR amplification of probe template from LA1589 genomic DNA. Purified PCR fragments from *SFT* genomic DNA were labelled by DNA 3′ End Biotinylation Kit (Pierce, Waltham, Massachusetts, USA). The binding reactions were conducted at room temperature in binding buffer [10 mm Tris (pH7.5), 50 mm KCl, 1 mm DTT, 2.5% glycerol, 0.05% NP‐40, 5 mm MgCl2, 0.5 mm EDTA, 50 ng/mL poly (dI·dC)] containing 1.5 μg purified GST‐SlZFP2 fusion protein and 50 fmol probes. Protein–DNA complex was separated on 6% native polyacrylamide gel in 0.5xTBE and then was transferred onto Hybond‐N+ nylon membrane (GE Healthcare Life Sciences, Chicago, USA). Protein–DNA interaction was detected using Light Shift Chemiluminescent EMSA Kit (Thermo Fisher Scientific, Waltham, Massachusetts, USA).

### Transient gene expression in *N. benthamiana* leaves and *Arabidopsis* protoplasts

For transient gene expression in *N. benthamiana* leaves, the reporter construct for expression of the YFP‐SFT fusion protein driven by the native *SFT* promoter was made by placing the 2.0‐kb *SFT* promoter amplified using primer XP2845 and XP1038 to the upstream of the *YFP‐SFT* coding sequence, which was prepared by cloning the full‐length cDNA of *SFT* amplified using primers XP1830 and XP2849 to the 3′ end of the *YFP* coding sequence (information of all primers used in this study can be found in Table S3). The expression cassette was then cloned into the pZH001 vector derived from pBI121. The two constructs for expression of *SlZFP2* (effector) and *YFP* (control) under the control of the *35S* promoter have been described previously (Weng *et al*., 2015). After verification by sequencing, the plasmids were introduced into the *Agrobacterium tumefaciens* strain GV3101, respectively. *A. tumefaciens* GV3101 strain containing reporter or control plasmid was co‐infiltrated with the agrobacteria containing the effector plasmid into *N. benthamiana* leaves. After three days postinfiltration, transient expression of YFP‐SFT fusion protein was monitored using an Olympus FV1000 confocal microscope. Transient gene expression in *Arabidopsis* protoplast was conducted as described previously with a plasmid containing GUS expression cassette driven by 1.8‐kb *SFT* promoter amplified by primers XP1773 and XP2070 (Weng *et al*., 2015).

## Supporting information


**Figure S1** Positive regulation of *SFT* expression by *SlZFP2* in mature green fruits.
**Figure S2** Flowering time and *SFT* expression in the leaves of ABA‐deficient mutants and their wild types.

**Table S1** Differentially expressed genes in p35S:HA‐SlZFP2 lines and nontransgenic controls.

**Table S2** Comparison of transcript levels determined by RNA‐seq.

**Table S3** Information of primers used in the study.
