# Supplementary material for: Manipulation of flowering time and branching by overexpression of the tomato transcription factor SlZFP2
Source: Plant Biotechnol J. 2016 Jun 29;14(12):2310–21. doi: 10.1111/pbi.12584 (PMC5103233; doi:10.1111/pbi.12584)
Supplement: Supplementary file 1 — Figure S1 Positive regulation of SFT expression by SlZFP2 in mature green fruits. Figure S2 Flowering time and SFT expression in the leaves of ABA‐deficient mutants and their wild types. [file PBI-14-2310-s003.doc]

**
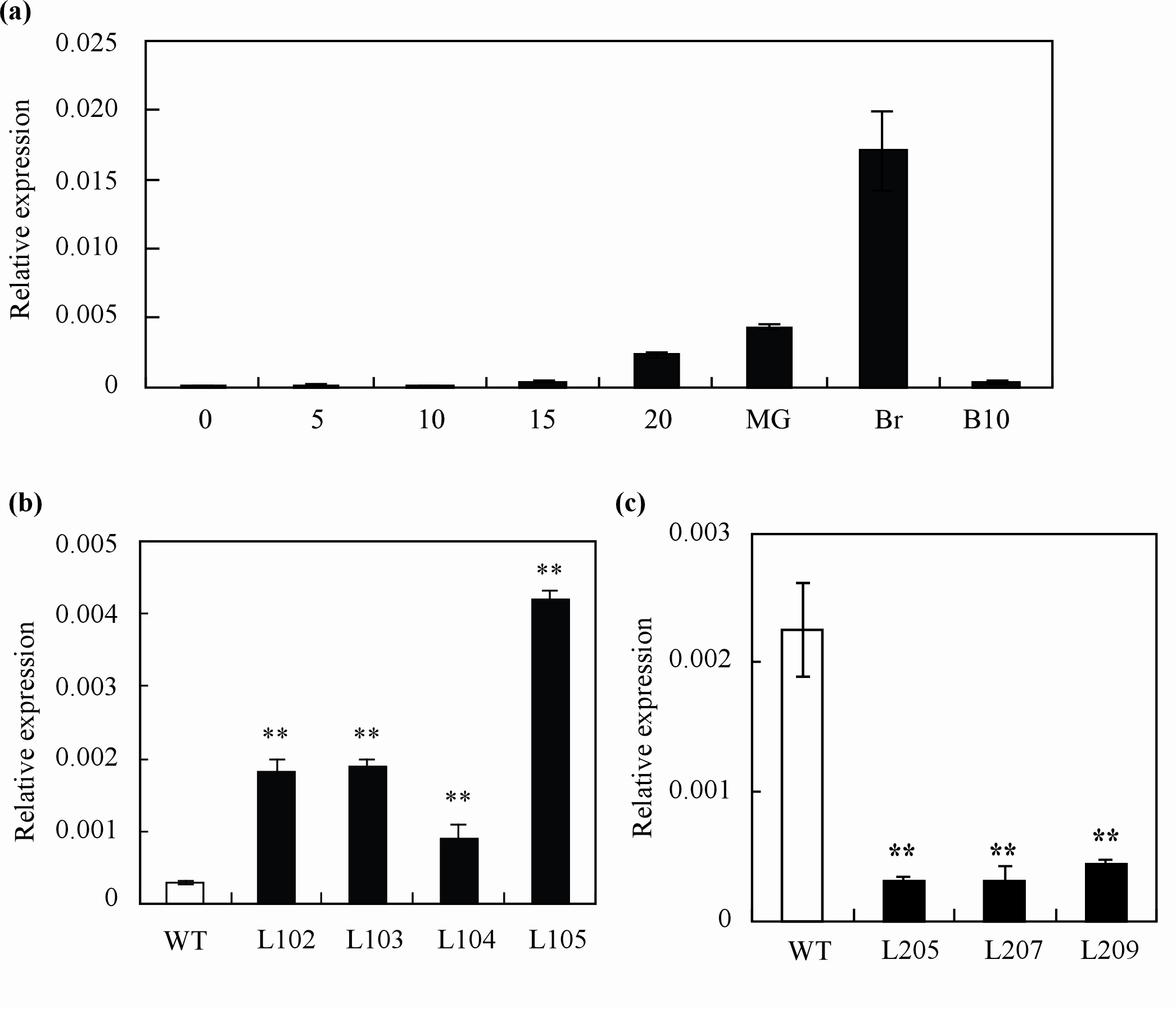
**

**Figure S1. Positive regulation of *SFT* expression by *SlZFP2* in mature green fruits**

(**a**), Transcript levels of *SFT* during fruit development and ripening. (**b**), Transcript levels of *SFT* in the mature green fruits of four *HA-SlZFP2* overexpression lines. (**c**), Transcript levels of *SFT* in the mature green fruits of three *SlZFP2* RNAi lines (L205, L207 and L209). Data were presented as mean ± SD of three biological replicates. Statistical significance of *p*-values was based on Student’s t-test. *, p<0.05; **, p<0.01.

**
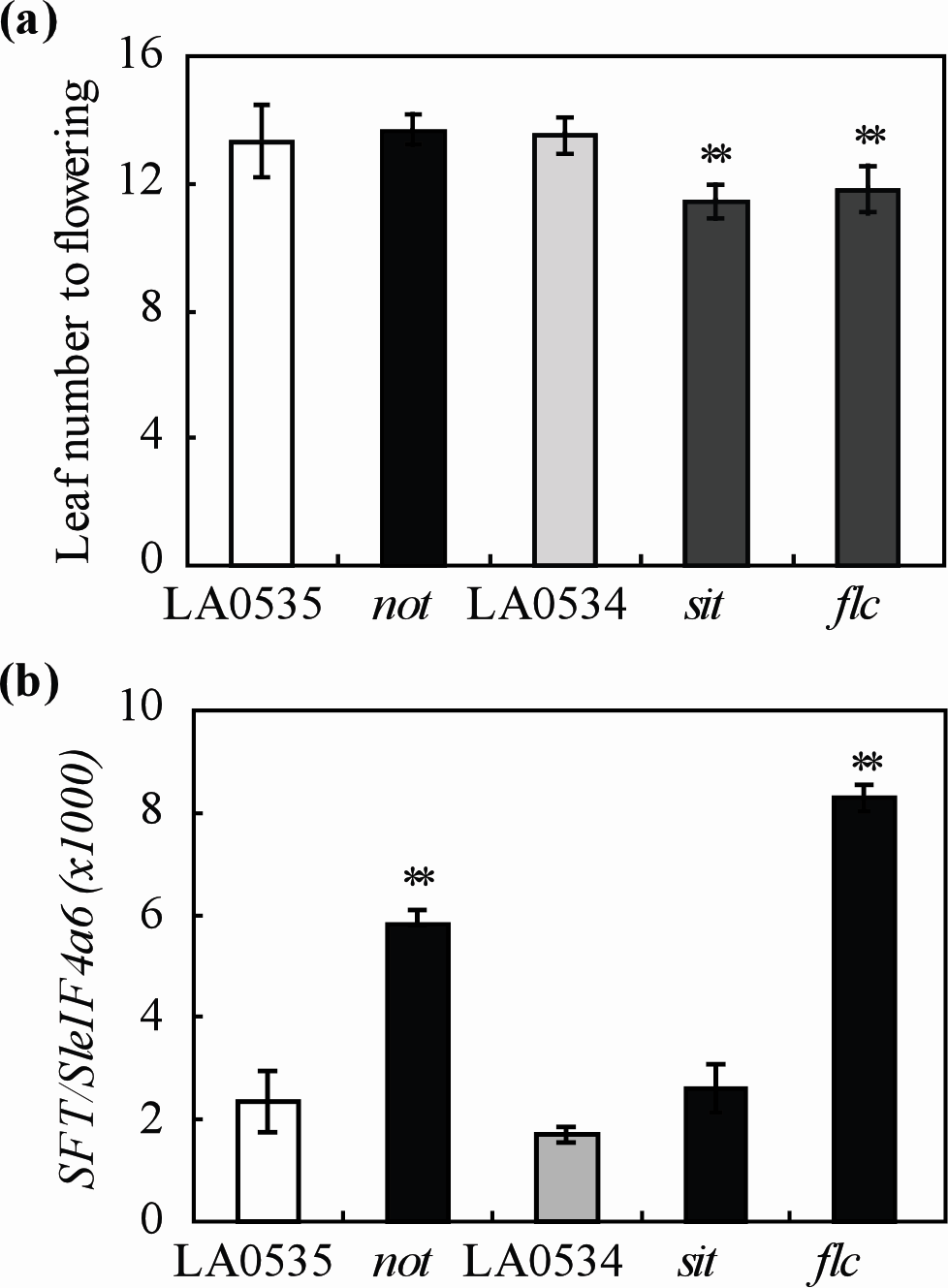
**

**Figure S2. Flowering time and *SFT* expression in the leaves of ABA deficient mutants and their wild types**

(**a**), Flowering time of three tomato ABA deficient mutants and their wild type controls. N=5-10. (**b**), *SFT* expression in the leaves of ABA deficient mutants and their wild type controls. Total RNA was extracted from fully expanded leaves. LA0535 is the wild type isogenic line of the *not* mutant, and LA0534 is the wild type isogenic line of *sit* and *flc*. Data were presented as mean ± SD of three biological replicates. Statistical significance of *p*-values was based on Student’s t-test. *, p<0.05; **, p<0.01.
